# Elderly people in humanitarian crises, a forgotten population: A call for action

**DOI:** 10.1371/journal.pgph.0002142

**Published:** 2023-07-17

**Authors:** Elburg van Boetzelaer, Joyce L. Browne, Sonali Vaid, Umberto Pellecchia, Judith van de Kamp, Oscar H. Franco, Amrish Y. Baidjoe

**Affiliations:** 1 Médecins sans Frontières, Operational Centre Amsterdam, Amsterdam, The Netherlands; 2 Global Public Health & Bioethics, Julius Centre for Health Sciences and Primary Care, University Medical Centre Utrecht, Utrecht University, Utrecht, Netherlands; 3 Incluve Labs, New Delhi, India; 4 Médecins sans Frontières, Operational Centre Brussels, Brussels, Belgium; 5 Médecins sans Frontières, Luxembourg Operational Research Unit, Luxembourg City, Luxembourg; 6 London School of Hygiene and Tropical Medicine, Department of Infectious Disease Epidemiology, London, United Kingdom; PLOS: Public Library of Science, UNITED STATES

The globally growing ageing population, an increasing number of natural disasters, and the ongoing effects of climate change will result in more complex humanitarian crises in the decades to come [1]. The proportion of the population aged 50 and over in fragile countries, where conflict and disasters are more likely to cause greatest harm, is expected to rise from 12.3 per cent (219.9 million) in 2020 to 19.2 per cent (586.3 million) in 2050 [2]. UNHCR warns that many challenges associated with ageing will surface earlier (i.e. before 60 years of age) in populations that have experienced trauma, prolonged poor nutrition status and exposure to disease [3]. Therefore, the specific needs of ‘older people’ are increasingly context-appropriate, which may mean going beyond defining people by their age.

Humanitarian crises tend to expose, and further widen, pre-crises inequalities and inequities and exacerbate existing vulnerabilities. The projected increase of weather-related humanitarian emergencies will disproportionately affect vulnerable groups, especially older people, who may have pre-existing health issues and nutrition status that limits mobility, physical functioning and access to health services. Their typically higher dependency on social and financial support networks in daily life make elderly people even more vulnerable [2]. During humanitarian crises, pre-existing conditions can be further exacerbated as older people are less likely to be able to relocate and therefore at may separate from their families and support networks. In addition to that, they risk a lack of access to health services, clean water and appropriate food. This puts them at increased risk of poor health outcomes including disability, injury, malnutrition and mental health issues [1, 4]. ‘Elderly people’ are not a homogenous group, and experience additional vulnerability factors such as older age, female gender, being widowed, increased exposure to traumatic events, prior mental health problems, low income and education and rural residency [4]. For example, women living in humanitarian context are disproportionally affected by humanitarian crises, exacerbating pre-existing gender norms and inequalities [5].

## Lacking age-inclusive humanitarian assistance leaves voices unheard and unaccounted for

Despite the existence of several guidance documents on age-inclusive humanitarian assistance [6, 7], older people are seldom included in the design, implementation and evaluation of humanitarian interventions, leaving their voices unheard and their needs likely largely unaccounted for [8]. This is problematic for several reasons.

First, ensuring appropriate, accessible and inclusive humanitarian assistance for all is a basic human right and an ethical imperative according to the fundamental humanitarian principles of humanity and impartiality [9]. In humanitarian crises, decision-making is typically based on where we think most impact can be attained. In humanitarian organizations, “impact” is typically defined through a westernized lens that views impact as saving lives that can be economically productive through contributing to a workforce. This utilitarian-based decision-making, in which maximizing economic well-being at a societal level is prioritized, inadvertently deprioritizes older people. For example, the COVID-19 pandemic painfully exposed age-discrimination around the world when it came to the distribution of scarce goods such as intensive care services and vaccines [10]. However, despite these principles, in the reality of the global health landscape there are only a few actors that address the specific needs of older people in humanitarian emergencies, while most are less considerate of the vulnerability of this sub-group and focus on the general population instead.

Second, studies and needs assessments including or specifically focusing on older people are scarce [11]. The lack of data on the specific needs of older people in humanitarian crises detriments inclusive, evidence-based humanitarian assistance and leaves it often inadequate and inappropriate for older people. For example, shelters, water supplies, toilets, food distribution points and health centres are rarely designed to be accessible for people with disabilities or limited mobility [12]. Older people are often left out of communication networks, due to limited access to or use of digital devices. Thus, they may not receive adequate information during a crisis and are deprived of support networks that increasingly emerge online in crises situations [13].

Third, older people often have unique skills, knowledge and experiences that come from many years lived in locations that are prone to disaster. When knowledge held by people who belong to marginalized groups, such as elderly people, is systematically afforded less credibility and if their interpretive resources are not recognized, this can be labelled as *an epistemic wrong* [14]. Epistemic wrongs are moral wrongs that occur in processes involved in knowledge production, use or circulation. The systematic exclusion of elderly people leads to *epistemic injustice* if elderly people are unable to produce, use or circulate their knowledge because knowledge is produced, used or circulated in isolation from them. In addition, older people often have a key role in family structures and as community connectors. They often have a position of respect and have a leadership role in their communities [15]. When given the chance, older people can be uniquely positioned to contribute to emergency preparedness, response and recovery. In addition to their position in the community, older people tend to hold community knowledge as they have lived experience of what has worked and not worked in the past [9]. Including older people in the different phases of the humanitarian program cycle will contribute to interventions that are more context-appropriate and are owned and supported by communities.

## Call for action: Inclusion of elderly people in all phases of humanitarian program cycle

**Fig 1:**
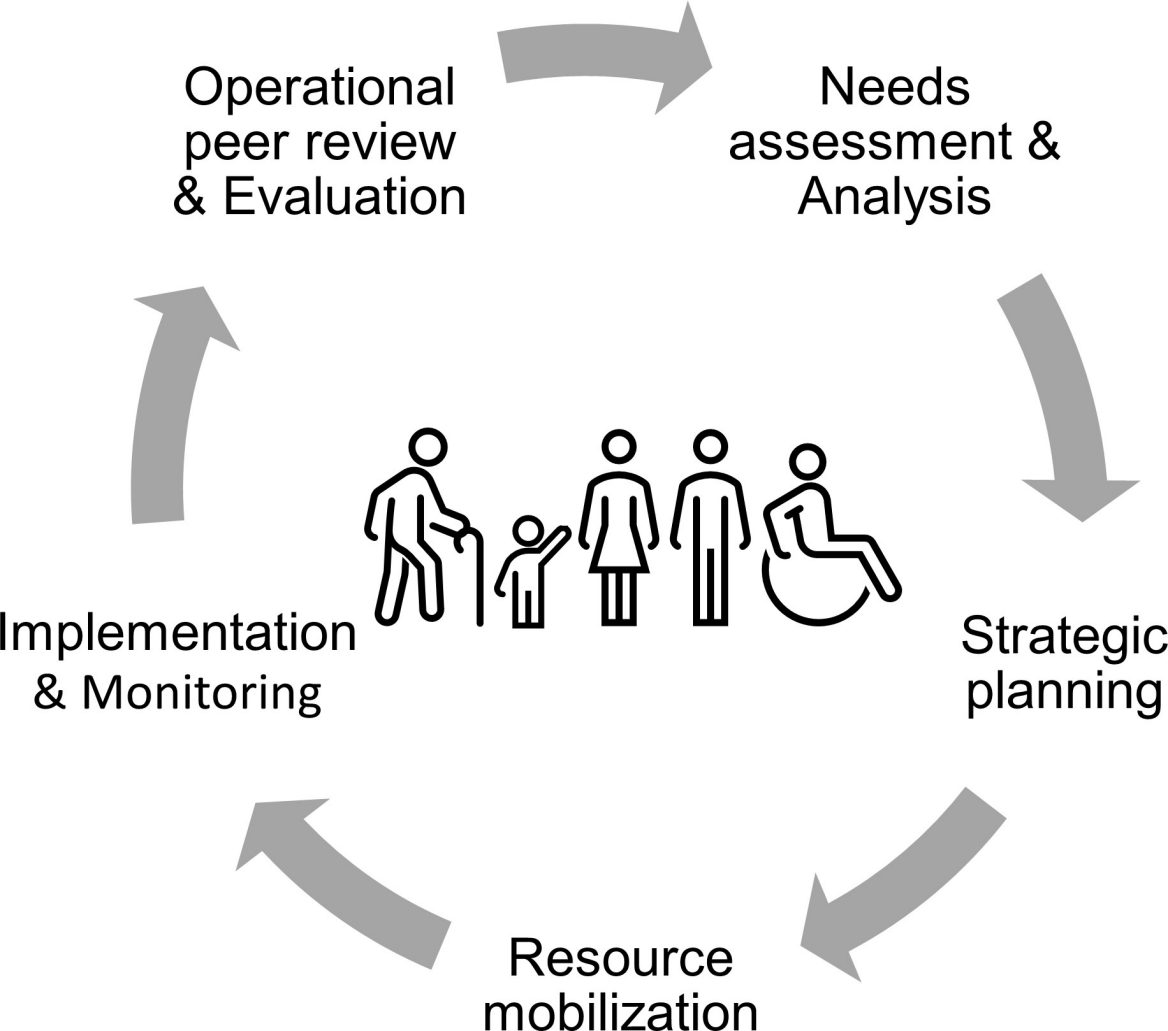
Humanitarian program cycle.

For all these reasons, we call for more emphasis on the inclusion of older people in all phases of the humanitarian program cycle (Fig 1). It is essential that organizations involved in the humanitarian crises move proactively towards action and cooperatively commit to explore and implement approaches to better assess and address the needs of older people in humanitarian emergencies, include these in decision-making and push for advocacy to consider older people’s needs within the humanitarian space. We concretely propose that all actors involved in emergency preparedness, response and recovery undertake conscious, deliberate and structured efforts. And in doing so, to start by ensuring that the definition of ‘older people’ is context-appropriate and community-driven, which may mean going beyond defining people by their age. Subsequently, to ensure inclusion of older people in data collection in humanitarian emergencies allows disaggregated data analysis to get a better understanding of specific needs and gaps in services and to strengthen the evidence base on health needs of older people in humanitarian emergencies. And most importantly, to learn from older people, their lived experience, and insights as those will contribute to more adequate and appropriate emergency preparedness, response and recovery. Only if we as humanitarian actors include elderly people in the planning, implementation and evaluation of health programs, do we live up to the ethical imperative of providing appropriate and accessible humanitarian assistance for all as a basic human right.
